# Eighteen-Year Follow-Up of Bone Sculpting Using the 3A-2B Biological Rule for Immediate and Late Implant Placement With Immediate Loading in Maxillary Fixed Prostheses: A Clinical Report

**DOI:** 10.1155/crid/6660284

**Published:** 2025-06-25

**Authors:** Fernando Rojas-Vizcaya

**Affiliations:** ^1^Department of Prosthodontics, University of North Carolina, Chapel Hill, North Carolina, USA; ^2^Department of Prosthodontics, Guangxi Medical University College & Hospital of Stomatology, Nanning, Guangxi, China

**Keywords:** bone sculpting, craniofacial growth, fixed prosthesis, immediate loading

## Abstract

Partial edentulism requiring maxillary rehabilitation with dental implants, whether using segmented or splinted fixed prostheses, often involves three-dimensional tooth position planning. This planning can lead to crown margins that either contact or encroach upon the remaining alveolar bone, a consequence of previous supraeruption. Supraeruption is associated with periodontal growth, where the periodontal ligament and bone develop concurrently with the erupting tooth. For successful outcomes in such cases, it is crucial to perform bone recontouring before implant placement, creating adequate space for the biological width, which is essential for achieving a segmented, implant-supported, fixed prosthesis. In the patient described in this case report, who received eight implants and four fixed partial prostheses, peri-implantitis required the removal of a posterior implant after 18 years of prosthesis use, necessitating retreatment in the corresponding quadrant. Notably, interproximal contacts between the prostheses had opened, likely due to continued craniofacial growth and bone remodeling, although this did not affect the patient's functionality. The recontoured bone and soft tissues maintained their position, preserving both the interproximal papillae and gingival contour in the esthetic zone.

## 1. Introduction

Immediate placement and loading protocols for complete maxillary arch rehabilitation are well documented and predictable, with high success rates [1]. Treatment planning for patients requiring complete, implant-supported, fixed prostheses begins by determining the three-dimensional tooth positioning of naturally sized maxillary central incisors (MCIs) in relation to the patient's face and lips with the correct vertical dimension [2]. Subsequently, the distance between the margins of planned clinical crowns (MPCCs) and the remaining bone should be analyzed [3]. If the distance is 3 mm, corresponding to the space required for the biological width [4], the implant must be placed at the bone level, and a pink-free Fixed Prosthesis 1 (FP1) is obtained, with naturally sized teeth emerging from the patient's soft tissue (ST). When the distance exceeds 3 mm, longer teeth (e.g., > 12.5–13 mm) that emerge from the patient's ST may be chosen, replacing the crown and a portion of the root, for an FP2 prosthesis [5]. If the distance exceeds 3 mm and creating long teeth is not aesthetically acceptable, an FP3 prosthesis should be fabricated, featuring natural-sized teeth with simulated ST in the prosthesis [3, 5, 6]. In patients with unopposed teeth, supraeruption with periodontal growth can occur, where the periodontal ligament and bone develop together with the erupting tooth [7]. In these patients, the distance could be < 3 mm, indicating bone reduction for the FP1 prosthesis [3, 8–11], and the 3A-2B biological rule must be considered [11], which defines optimal 3D implant position as follows: The implant platform should be placed 3 mm apical from the MPCC for an adequate biological width (3A), and a minimum of 2 mm of remaining buccal bone must be maintained to prevent its resorption (2B). In patients with immediate implants, approximately 1 mm of bundle bone is lost as it is related to the presence of the tooth, and the buccal gap after implant placement must be 1.5–2 mm such that after bone remodeling, the buccal bone that covers the implant is 2 mm [12]. The use of implants with conical seal connections is recommended to minimize marginal bone loss [3]. Bone remodeling (BR) continues throughout the whole of life [13]. Some studies have shown continuous craniofacial growth (CCG) and BR in patients up to 62 ^14^ or, in anthropometric measurements, transversal growth in subjects between 16 and 90 years of age [14].

Therefore, its implications for the long-term outcome of implant-supported prostheses must be considered, because unlike in implants, physiologic tooth migration can create open contacts mesial to single implants or implant-supported fixed partial dentures (FPDs) [15]. However, to the best of the author's knowledge, no long-term reports of open contacts between segmented FP1 implant-supported prostheses exist. Herein, a protocol involving bone reduction performed prosthetically to obtain FP1 prostheses, with results obtained after 18 years, has been described.

## 2. Case Presentation

**Figure 1:**
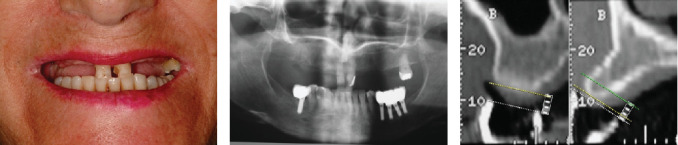
(a) Previous poor esthetic and functional condition. (b) Radiograph showing enough bone for implant placement. (c) Left, bone at 3 mm from margin; right, bone at 1 mm from margin.

A 62-year-old woman presented to a prosthodontic office with a maxillary left second molar and remaining MCIs with a poor prognosis because of extensive caries (Figure 1a) and remaining mandibular anterior natural teeth and implant-supported prostheses of the posterior mandibular areas (Figure 1b). Approximately two decades earlier, when the patient was in her 40s, another dentist had extracted her posterior mandibular teeth. Over the following 10 years, she experienced supraeruption of the opposing maxillary teeth and gingival growth. Subsequently, when the patient was aged 50 years, the same dentist placed bilateral posterior mandibular implants and FPDs in the edentulous areas, which occluded with the extruded maxillary posterior teeth. Ten years later, when the patient was aged 60 years, the previous dentist extracted her extruded maxillary posterior teeth bilaterally, resulting in the residual overgrown bone that needed recontouring [7]. The patient signed an informed consent form permitting the use of her information and photographs. The maxillary left second molar maintained the vertical dimension against the implant-supported mandibular FPD; a free space of 2 mm was observed. Therefore, modifications were not necessary in the vertical dimension but only in the overgrown bone. The existing mandibular implants were at the same bone level as when first implanted, and the anterior teeth had no periodontal problem. Therefore, existing implants were utilized to fabricate an FPD spanning from the left second premolar to the second molar. Additionally, an implant in the position of the right first molar supported a screw-retained crown. No restorative treatments were performed on the remaining anterior mandibular teeth. A fixed prosthodontic treatment, with immediate dental implant placement in both MCIs, six implants in the healed ridge, and immediate loading prosthesis for the eight implants, was planned. The clinical crown lengths of the two MCIs were 8 mm each, and 1 mm of the MCIs could be observed with the lip at rest having a 22-mm lip length; therefore, these two MCIs were planned to be elongated by 1 mm in the incisal direction and 1.5 mm in the apical direction to obtain an average size of 10.5 mm. Thus, 2 mm of the MCIs would be observed with the lip at rest. Based on this information, a diagnostic wax-up was conducted. Subsequently, a thermoplastic template (Temp Splint 0.5 mm; Denta Flux, Madrid, Spain) was fashioned on a duplicate cast of the wax-up, which was then converted into a radiographic template. Twelve radiopaque markers, crafted from 1 mm lead strips sourced from periapical radiograph films, were affixed using sticky wax (Kerr Corporation, Orange, California) from the buccal to the palatal clinical crown margin of each intended crown. Following the completion of computerized tomography (CT), the lead strips were removed, and the thermoplastic template was adapted into a surgical guide. The MPCCs were delineated with an indelible marker to enhance margin visibility, while perforations and vertical lines were created in areas earmarked for implant placement to aid in bone reduction, milling, and orientation. Moreover, the guide extended across the palate to encompass the remaining left maxillary second molar, facilitating its positioning and stabilization during surgery.

**Figure 2:**
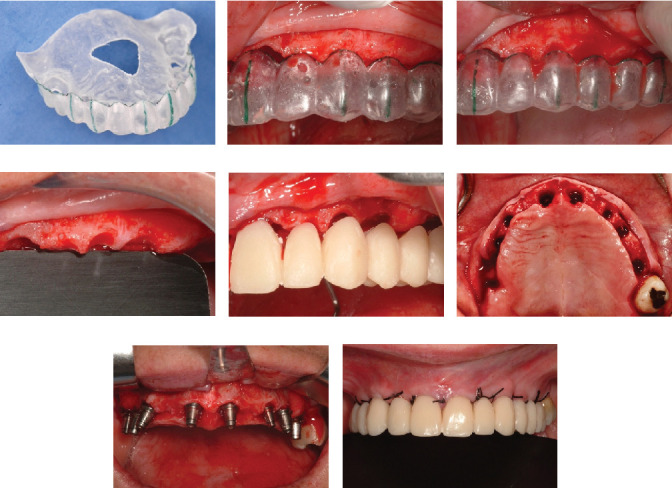
(a) Surgical guide utilized for bone reduction and implant placement, featuring palatal support and perforation for fit evaluation. (b) Template before bone sculpting showing bone inside crowns. (c) Template after bone reduction showing 3-mm gap between margins and bone. (d) Bone sculpting and maintenance of corresponding interproximal bone for papilla support. (e) Provisional restoration showing band of free space below margin of crowns. (f) Occlusal view showing gap of 1.5–2 mm in both maxillary central incisors. (g) Abutment finished line in both maxillary central incisors at level of bundle bone. (h) Immediate loading cement-retained full-arch prosthesis.

**Figure 3:**
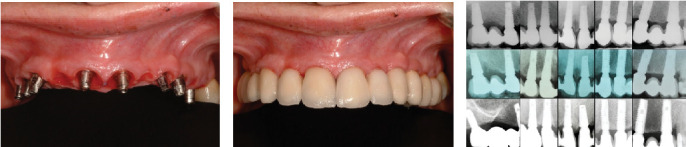
(a) Soft tissue sculpted after 12 weeks. (b) Definitive cement-retained fixed porcelain fused to metal partial dentures with sculpted soft tissue at day of cementation. (c) Periapical radiographs at delivery time (top), 10-year (center) and 18-year (bottom) follow-up findings showing maintenance of bone at level of implants and interproximal bone without modifications.

**Figure 4:**
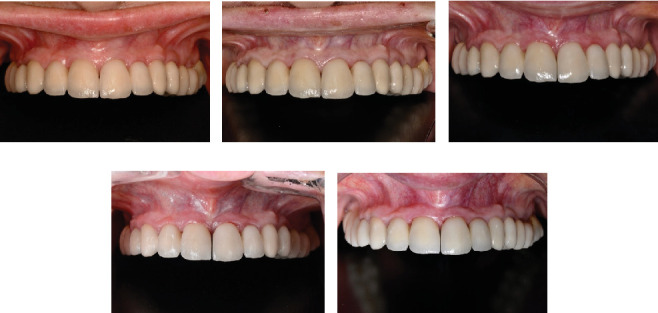
(a) After 1 year of follow-up. (b) After 5 years of follow-up. (c) After 10 years follow-up, small gap was visible between prostheses (d). After 15 years follow-up. (e) After 18 years follow-up, small diastema is visible, contoured soft tissue was maintained, and inerproximal papillae filled all embrasures.

Following the protocol described by Rojas-Vizcaya in 2012 [3], eight maxillary implants were planned; the positions are described in Table 1. Four FPDs, extending from the MCI to the canine and first premolar to the first molar on each side, were planned. The decision to fabricate four FPDs was based on the application of the segmentation concept. This approach allows that, in the event of an implant failure or a complication with a specific prosthesis, only the problematic implant or prosthesis can be removed and addressed without the need to disturb the other prostheses. This strategy offers greater ease in maintenance and management of potential complications, preserving the integrity of the remaining prostheses and reducing the need for more extensive interventions [3]. Radiographically, the distance between the facial MPCCs and the remaining bone could be measured (Figure 1c) and is presented in Table 1. On the day of surgery, after dental extractions and raising a full-thickness flap from molar to molar, the thermoplastic guide (Figure 2a) was positioned (Figure 2b), and the bone was sculpted using a 2-mm-diameter round surgical bur until a 3-mm distance between the MPCCs and bone was obtained (Figure 2c). The reduction was done from the buccal to the palatal side to avoid contact with the abutment shoulder, without reducing the interproximal bone to provide support to the interproximal papillae (Figure 2d). The space was evaluated by positioning the prosthesis (Figure 2e). Subsequently, eight fluoride-modified screw-shaped implants (Fixture MT OsseoSpeed; Astra Tech AB) were placed according to the 3A-2B biological rule. In the extraction sockets, the implants were at least 1.5 mm below the buccal bone level and always 3 mm away from the MPCCs, without reduction of the bundle bone since 1 mm is expected to be lost [12], and a 1.5–2 mm jumping distance was maintained (Figure 2f) to obtain 2 mm of remaining buccal bone after remodeling [12]. Eight cement-retained abutments (direct abutments; Astra Tech AB) were then screwed into the implants (Figure 2g). The flap was sutured using 3-0 silk (Silk; Stoma). To deliver the provisional restoration, the protocol described by Rojas-Vizcaya in 2012 was followed [3] (Figure 2h). After 12 weeks, the ST acquired the shape of the provisional restorations (Figure 3a), which were then removed, and an abutment-level impression was made. Four metal-ceramic FPDs were made in the laboratory and cemented using temporary cement (Temp-Bond; Kerr Italy S.r.l.) (Figure 3b). The fit of the FPDs over the abutments was verified on periapical radiographs, and the marginal bone at the implant level was confirmed (Figure 3c top). Canine guidance was the type of occlusion provided. The patient underwent annual checkups over 18 consecutive years. At the 8-year follow-up, a slight interproximal contact loss was observed between the two MCIs and the canines and maxillary first premolars on both sides, probably because of craniofacial growth and bone remodeling. In the following 9 years, a small gap in those areas became more evident but imperceptible to the patient without causing esthetic or functional discomfort (Figure 4a). At the 10-year follow-up, the right maxillary first molar implant was treated for peri-implantitis (Figure 3c center) using a nonsurgical approach but was removed 6 years later. A new implant was placed in the area of the right maxillary second molar (OsseoSpeed EV; Astra Tech; Dentsply Sirona), and a new cement-screwed fixed prosthesis was made using monolithic zirconia from the maxillary first premolar to the maxillary second right molar. At the 18th year of follow-up, in all other areas, the interproximal bone was observed at the implant level, the bone peak between both MCIs and other teeth was maintained (Figure 3c bottom), and the ST remained stable (Figure 4b) with the same esthetics as those in the first year. With the new prosthesis, the open contact between the right maxillary canine and the right first premolar was closed. As the other two open contacts did not cause any esthetic or functional concerns for the patient in the last 9 years, the other prostheses were not changed.

## 3. Discussion

Patients with FP1 of the entire arch, segmented and completed with surgically sculpted bone using various protocols, such as analog [3], hybrid [8], or digital [9, 10] techniques, have reportedly achieved initial optimal esthetic success. The 3A-2B biological rule [11] has been consistently applied in these cases, ensuring the creation of the necessary space for the biological width between the prosthesis and the bone. The initial esthetic outcomes observed in this case report align with those found in previous short-term reports [8–10]; however, it is important to note that none of these reports included long-term follow-up. As such, the results from monitoring the patient over an 18-year period in this case cannot be directly compared to the findings of these previous studies, nor can the interproximal contact opening achieved using fixed prostheses be compared to those in cases where segmented prostheses were used [3].

To the best of the author's knowledge, there are no existing publications addressing the space created between fixed implant-supported segmented prostheses in the short- or long-term following complete maxillary rehabilitation. A comparison of the advantages and disadvantages of segmented and cemented FP1 prostheses with those of complete arch and screwed FP1 prostheses has been made in the literature. Segmented prostheses allow for the prompt treatment of problematic implants without the need to remove other prostheses, as demonstrated in this case. Additionally, cementation effectively conceals the screw access hole, especially when located vestibularly. However, the use of eight implants to support four FP1 prostheses is required [3]. Cemented prostheses, while beneficial in some aspects, may present challenges such as difficulty in removal and potential for decementation over time. Furthermore, cement traces are not visible, potentially leading to peri-implantitis.

For these reasons, the use of complete and screwed FP1 prostheses may be advisable in certain situations. However, complete FP1 prostheses come with risks, as the use of four implants [8] implies that the failure of any one implant would necessitate the replacement of the entire prosthesis. Additionally, the potential loss of interproximal papillae could result in the formation of black triangles if interproximal bone loss occurs. Thus, the use of implants with internal conical connections, which minimize marginal bone loss, is essential [3]. A remaining 2 mm of buccal bone is considered crucial for preventing resorption. However, long-term studies are lacking to demonstrate whether this bone remains intact or resorbs slowly over the course of the patient's life. Resorption could result in esthetic complications, such as the appearance of gray ST or vestibular recession, particularly in patients with a gummy smile.

In these cases, the treatment approach should be evaluated carefully, considering the patient's age, satisfaction with the prosthesis, and whether bone resorption is present without peri-implant disease. Prosthesis separation and the loss of interproximal contacts may result in small diastemas, typically without evidence of occlusal trauma. These phenomena can be attributed to CCG and BR [13, 14, 16]. According to Velemínská et al. (2021), craniofacial morphology continues to change throughout adulthood due to slow, continuous appositional growth, resorption, and remodeling [13]. In a study conducted by Al-Tai et al. (2022), the maxilla length, measured from the anterior to the posterior nasal spine, was shown to grow up to the age of 62 [16], which corresponds to the age at which implants were placed in the patient of this report. However, there was no long-term follow-up in this study to assess whether the maxilla continued to grow beyond this age. In contrast, Farkas et al. (2004) reported an increase in transversal measurements of the maxilla in individuals aged 60 to 90 years [14].

Based on these findings, the diastemas observed between the FPDs in this patient are likely due to the combined effects of CCG and BR occurring between the patient's age of 62 and 80. However, additional studies are needed to fully understand these mechanisms and their impact on the long-term outcomes of maxillary rehabilitation with implant-supported prostheses.

## 4. Conclusion

For patients requiring fixed implant-supported prostheses, the application of the biological rule 3A-2B can aid in determining the need for bone reduction to position implants 3 mm from the MPCC. By maintaining 2 mm of buccal bone, it is possible to preserve the bone, gingival, and interproximal papillae levels within the aesthetic zone for over 18 years. Considering the patient's age, opting for a full-arch prosthesis over segmented ones is advisable to mitigate the risk of prosthesis separation due to CCG and BR. Utilizing screw-retained prostheses instead of cemented ones is recommended to enhance access for peri-implant treatments during the initial stages, consequently reducing the number of implants needed to support a single prosthesis and facilitating patient hygiene. It is further recommended to conduct long-term studies to assess the impact of CCG and BR on comprehensive full-mouth rehabilitation outcomes.

## Figures and Tables

**Table 1:** Analysis of physical factors that influence implant placement in maxillary arch.

**Maxillary prosthetic tooth**	**Prosthetic item**	**Crown margin/bone distance**	**Surgical procedure required to obtain 3A**	**Surgical procedure required to obtain 2B**	**Implant diameter/length (mm)**
Right first molar	Implant	3.0	Implant placement at bone level	Palatal position keeping 2 mm of the buccal bone	5.0/9
Right second premolar	Ovate pontic	2.5	0.5-mm bone reduction	N/A	N/A
Right first premolar	Implant	1.0	2.0-mm bone reduction	Palatal position keeping 2 mm of the buccal bone	4.5/11
Right canine	Implant	1.0	2.0-mm bone reduction	Palatal position keeping 2 mm of the buccal bone	4.5/11
Right lateral incisor	Ovate pontic	3.0	None	N/A	N/A
Right central incisor	Implant	1.5	Implant placement 1.5 mm below the bone	Maintain a 1.5–2-mm gap	4.5/11
Left central incisor	Implant	1.5	Implant placement 1.5 mm below the bone	Maintain a 1.5–2-mm gap	5.0/9
Left lateral incisor	Ovate pontic	0.5	2.5-mm bone reduction	N/A	N/A
Left canine	Implant	0.5	2.5-mm bone reduction	Palatal position keeping 2 mm of the buccal bone	3.5/11
Left first premolar	Implant	1.5	1.5-mm bone reduction	Palatal position keeping 2 mm of the buccal bone	4.0/11
Left second premolar	Ovate pontic	3.0	None	N/A	N/A
Left first molar	Implant	3.0	Implant placement at the bone level	Palatal position keeping 2 mm of the buccal bone	5.0/11

Abbreviation: N/A, not applicable.

## Data Availability

Data sharing does not apply to this article as no new data were created or analyzed in this study.
